# Children’s Oxygen Administration Strategies And Nutrition Trial (COAST-Nutrition): a protocol for a phase II randomised controlled trial

**DOI:** 10.12688/wellcomeopenres.17123.1

**Published:** 2021-09-03

**Authors:** Sarah Kiguli, Peter Olopot-Olupot, Florence Alaroker, Charles Engoru, Robert O. Opoka, Abner Tagoola, Mainga Hamaluba, Hellen Mnjalla, Ayub Mpoya, Christabel Mogaka, Damalie Nalwanga, Eva Nabawanuka, James Nokes, Charles Nyaigoti, André Briend, Job B. M. van Woensel, Richard Grieve, Zia Sadique, Thomas N. Williams, Karen Thomas, David A. Harrison, Kathryn Rowan, Kathryn Maitland

**Affiliations:** 1 Paediatrics, Makerere University, Kampala, Uganda; 2 Paediatrics, Mbale Regional Referral Hospital, Mbale, Uganda; 3 Paediatrics, Soroti Regional Referral Hospital, Soroti, Uganda; 4 Paediatrics, Jinja Regional Referral Hospital, Jinja, Uganda; 5 Paediatrics, Kilifi County Hospital, Kilifi, Kilifi, POBox230, Kenya; 6 KEMRI Wellcome TRust Research Programme, Kilifi, Kilifi, POBox230, Kenya; 7 School of Medicine, University of Tampere, Tampere, Finland; 8 Paediatric Intensive Care Unit, Emma Children’s Hospital and Academic Medical Center, Amsterdam, The Netherlands; 9 Centre for Statistical Methodology, London School of Hygiene & Tropical Medicine, London, UK; 10 Department of Infectious Disease and Institute of Global Health and Innovation, Imperial College London, London, UK; 11 Intensive Care National Audit, London, WC1V 6AZ, UK

**Keywords:** Children, Africa, Pneumonia, Randomised controlled trial, nutritional support, Ready to use Therapeutic Feeds, anthropometry, pathogen diagnosis

## Abstract

**Background: **To prevent poor long-term outcomes (deaths and readmissions) the integrated global action plan for pneumonia and diarrhoea recommends under the ‘Treat’ element of Protect, Prevent and Treat interventions the importance of continued feeding but gives no specific recommendations for nutritional support. Early nutritional support has been practiced in a wide variety of critically ill patients to provide vital cell substrates, antioxidants, vitamins, and minerals essential for normal cell function and decreasing hypermetabolism. We hypothesise that the excess post-discharge mortality associated with pneumonia may relate to the catabolic response and muscle wasting induced by severe infection and inadequacy of the diet to aid recovery. We suggest that providing additional energy-rich, protein, fat and micronutrient ready-to-use therapeutic feeds (RUTF) to help meet additional nutritional requirements may improve outcome.

**Methods:** COAST-Nutrition is an open, multicentre, Phase II randomised controlled trial in children aged 6 months to 12 years hospitalised with suspected severe pneumonia (and hypoxaemia, SpO _2_ <92%) to establish whether supplementary feeds with RUTF given in addition to usual diet for 56-days (experimental) improves outcomes at 90-days compared to usual diet alone (control). Primary endpoint is change in mid-upper arm circumference (MUAC) at 90 days and/or as a composite with 90-day mortality. Secondary outcomes include anthropometric status, mortality, readmission at days 28 and 180. The trial will be conducted in four sites in two countries (Uganda and Kenya) enrolling 840 children followed up to 180 days. Ancillary studies include cost-economic analysis, molecular characterisation of bacterial and viral pathogens, evaluation of putative biomarkers of pneumonia, assessment of muscle and fat mass and host genetic studies.

**Discussion: **This study is the first step in providing an option for nutritional support following severe pneumonia and will help in the design of a large Phase III trial.

**Registration:** **ISRCTN10829073** (6 ^th^ June 2018) **PACTR202106635355751** (2 ^nd^ June 2021)

## Abbreviations

16S rDNA          16S ribosomal deoxyribonucleic acid

BIA                    bioimpedance analysis

COAST              Children’s Oxygen Administration Strategies Trial

CRF                   Case Report Form

CTU                   Clinical Trials Unit

DFID                 Department for International Development

DMC                 Data Monitoring Committee

DNA                  deoxyribonucleic acid

EDCTP               European and Developing Countries Clinical Trials Partnership

HBSA                 Heterozygous Sickle Cell Trait

HbSS                 Homozygous Sickle Cell Haemoglobin

ICH-GCP           International Conference on Harmonization Good Clinical Practice

ICNARC            Intensive Care National Audit & Research Centre

KCH                  Kilifi County Hospital

KCTF                Kilifi Clinical Trial Facility

KEMRI              Kenya Medical Research Institute

KWTRP             KEMRI Wellcome Trust Research Programme

LRTI                  lower respiratory tract infection

MAM                 Moderate malnutrition

MRC                 Medical Research Council

MUAC               Mid-Upper Arm Circumference

ORC                   Receiver Operator Curves

PCR                  polymerase chain reaction

qPCR                specific quantitative polymerase chain reaction

RCT                  randomised controlled trial

RDT                  Rapid diagnostic test

REC                  Research Ethics Committee

RNA                 ribonucleic acid

RR                   relative risk

RSV                 Respiratory Syncytial Virus

RUFT                 Ready to use Therapeutic Feed

RUSF                  Ready to use Supplemental Feed

RV                   respiratory viruses

SAE                 serious adverse event

SAM                severe acute malnutrition

SCD                sickle cell disease

SIV                 site initiation visit

SP                   severe pneumonia

SpO _2_               oxygen saturation

TMG               Trial Management Group

TSC                Trial Steering Committee

TST                   Triceps skinfold thickness

UNICEF             United Nations Children’s Fund

VSP                very severe pneumonia

WAZ               Weight for age Z score

WGS               whole genome sequencing

WHO               World Health Organization

WHZ               Weight for height Z score

## Introduction

Pneumonia is the leading cause of childhood death in sub-Saharan Africa ^1^ however, short and long-term outcomes remain poor including death and hospital readmission ^2, 3^. To address long-term outcomes, the integrated Global Action Plan for Pneumonia and Diarrhoea developed by the World Health Organization (WHO) and United Nations Children’s Fund (UNICEF) recommend ‘continued’ feeding under the ‘Treat’ element of the Protect Prevent and Treat framework for pneumonia and diarrhoea interventions, but do not give any specific recommendations for nutritional support ^4^. Early nutritional support is commonly practiced in a wide variety of critically ill patients, including those with sepsis. Supportive nutrition is aimed at supplying vital cell substrates, antioxidants, vitamins and minerals, essential for normal cell function and to decrease hypermetabolism ^5, 6^.

### Risk factors for poor long-term outcomes in children with severe pneumonia

A prospective study exploring the survival of all children admitted to Kilifi County Hospital (KCH) found that mortality was higher in the post-discharge cohort than in the community cohort showing an increased hazard ratio for post-discharge mortality in children under 5 years with anthropometric evidence of undernutrition ^7^. Relevant to the question COAST-Nutrition trial seeks to address is children hospitalised with clinical diagnosis of very severe pneumonia (VSP), who had the second highest hazard ratio for post-discharge mortality (after malnutrition) of 4.1 (95%2.3-7.5) ^7^. This was supported by an additional study examining data from 2007 to 2021 involving 4184 children aged 1–59 months admitted KCH with severe pneumonia. Mortality and the risks for post-discharge mortality were examined in the year after discharge. In the 2279 children resident in the Kilifi demographic district who were followed up for survival, 70 (3.1%) died during 2163 child-years. Overall, 52% (95% confidence interval (CI) 37%, 63%) of post-discharge deaths were attributable to low mid-upper arm circumference (MUAC) ^8^.

### Supplementary feeding

The usual stress response to severe infection includes the increased release of catabolic hormones (cortisol and catecholamines) leading to a catabolic ‘hypermetabolic’ response in order to rapidly mobilise energy. The source is mainly via protein breakdown, leading to the conversion of amino acids (mainly alanine and glutamine) to glucose through liver gluconeogenesis. Consequently, there is rapid skeletal muscle breakdown leading to muscle cachexia or ‘wasting’ ^5^. This has been shown to have adverse consequences in the intensive care unit studies, even in previous healthy adults, delaying weaning from mechanical ventilation ^6, 9^. In children surviving pneumonia with normal nutritional status or for moderate acute malnutrition (MAM) there is no current recommendation for nutritional support therefore we have reviewed what is recommended for children with MAM in community programmes.

Supplementary feeding using ready to use supplementary feeds (RUSF) is often recommended for MAM. The nutrient intakes for optimal recovery of children with moderate acute malnutrition were reviewed in October 2008 at a joint consultation of the WHO, UNICEF, World Food Programme and the United Nations High Commissioner for Refugees in children under 5 years of age ^10^. The consensus view was that the desirable nutrient intakes in relation to energy were probably in the range between the recommended nutrient intakes for well-nourished children and the intakes recommended in the recovery phase for those with severe acute malnutrition(SAM) ^11^. For children with severe acute malnutrition (SAM) ready to use therapeutic feeds (RUTF) recommended a dose of 175–200 kcal/kg/day until they reach the nutritional discharge criterion (based on lack of oedema or MUAC >=12.5cm). However, since growth rates tend to slow towards the end of nutritional treatment ^12^ new strategies for management are now being considered. This includes a simplified and integrated SAM/MAM programme that use RUTF (rather than RUSF) and which has resulted in more favourable outcomes and is more pragmatic to current standard treatments ^13^.

Ready-to-use therapeutic food (RUTF) is a high-energy fortified food used for the treatment of SAM. During the period of SAM treatment RUTF paste is the sole source of food, except for breast milk in the case of breast-fed infants. It has also been used in the MAM programmes as a supplementary feed alongside of usual diet.

We considered the findings of the ComPAS study which recommended the integrated use of RUTF in MAM/SAM programmes and whether this could be extended to children at high risk of undernutrition/malnutrition. The ComPAS study suggested that for children with MAM (MUAC 11.5cm- < 12.5cm) 95% of children would receive 49% or more of their energy needs covered by the proposed protocol, with a median of 74% of their energy needs covered ^14^. The study by Maust *et al.* suggested this approach leads to superior outcomes ^13^. In the open-label Phase II randomised controlled COAST-Nutrition trial comparing supplementing usual diet with RUTF (intervention) compared to usual diet alone (control) ration 1:1 we aim to extend the use of RUTF to targeting children recovering from hospitalisation with severe pneumonia. The trial incorporates children across the anthropometric spectrum who are at high risk of needing nutritional support but excluding children with SAM (who will all receive RUTF at treatment doses). Our rationale considered that RUTF is widely used and if shown to benefit children following admission with pneumonia, could be rapidly deployed.

## Protocol

This trial is registered at ISRCTN (ISRCTN10829073, 6 ^th^ June 2018) and The Pan African Clinical Trials Registry (PACTR202106635355751, 2 ^nd^ June 2021). This is protocol version 4.0: date 17 ^th^ February 2020. This article is reported in line with the Standard Protocol Items: Recommendations for Interventional Trials (SPIRIT) guidelines ^15^. 

### Justification for the study

The high prevalence of pneumonia in children leading to poor outcomes including both short-term and long-term mortality and readmission post discharge constitutes a major public health challenge. This trial was designed to inform a key research gap on nutritional support and will provide preliminary evidence for health services in Africa on the basis of clinical effectiveness and costs.

### Our hypotheses

We propose that the excess post-discharge mortality associated with pneumonia may relate to the catabolic response and muscle wasting induced by severe infection and inadequacy of the diet to aid recovery ^16^.

## Objectives

### General objectives

Our principal objective is to establish whether supplementing feeding with RUTF will improve anthropometric outcomes and/or mortality to day 90 and day 180 in children hospitalised with severe pneumonia.

### Specific objectives

(i) To conduct a Phase II clinical trial comparing supplementary feeding for 56-days using RUTF in addition to usual diet in children aged 6 months or more admitted with severe pneumonia (but without severe acute malnutrition) versus usual diet alone (control) and to assess the impact on primary and secondary outcomes at 90-days and 180 days.
(ii) To describe the pathogenic aetiology of pneumonia using molecular diagnostics on stored blood samples and viral signatures from nasal flock swabs and effect on clinical outcomes.
(iii) Whether sickle cell disease determines outcome in children presenting with severe pneumonia and whether this is moderated by the nutritional intervention.
(iv) To assess relative muscle mass and fat mass at admission and overtime and whether reduced muscle mass is a key factor determining outcome in children suffering from pneumonia.
(v) To investigate whether a combination of clinical, point-of-care diagnostic tests, and/or biomarkers can more accurately identify children with severe pneumonia with culture-proven bacteraemia and/or chest X-ray evidence of consolidation.

## Methods

### Study sites

The trial will be conducted in four sites in two countries in Africa. In Uganda the trial will enroll children in Mbale Regional Referral Hospital, Soroti Regional Referral Hospital and Jinja Regional Referral Hospital. In Kenya, children will be enrolled at the Kilifi County Hospital. The trial will be overseen by KEMRI Wellcome Trust Research Programme (KWTRP) where the Trial Management Group (TMG) will be based and will coordinate the conduct of the trial.

### Study design

A Phase II multicentre open randomised controlled trial aimed to generate preliminary data on anthropometric evidence of growth and survival to day 90 in children receiving supplementary nutritional support in addition to usual diet (experimental) compared to usual diet alone (control).

### Study populations

Children will be considered eligible for enrolment in this trial if they fulfil all the inclusion criteria and none of the exclusion criteria.

### Inclusion criteria

• Aged between 6 months to 12 years
• History of respiratory illness (cough, upper respiratory tract symptom or any respiratory symptoms e.g. rapid breathing or increase work of breathing)
• Hypoxia (pulse oximetry reading of SpO _2_ <92% recorded in room air over 5 minutes)
• Any signs of severe pneumonia (from 2013 WHO clinical definitions for pneumonia) ^17^

### Exclusion criteria

• Severe malnutrition (MUAC <11.5cm)
• Consent refusal by parent/carer
• Previously recruited to COAST-Nutrition trial
• Known chronic lung disease (not including asthma) and cyanotic congenital cardiac disease

### Sample size determination

Mid-upper arm circumference (MUAC) has been selected as the primary criterion for nutritional recovery because it predicts mortality better ^18^, is less affected by oedema than other anthropometric measures and is also a good index of muscle mass. The primary endpoint will therefore be a composite of MUAC change from baseline (taken at admission to hospital) and mortality at 90 days. Patients will be ranked firstly by their survival status at day 90 (with death being the worst possible outcome), then by MUAC change in surviving patients, and the resulting ranks will be compared between arms using a two-sample rank-sum (Mann-Whitney) test with alpha of 0.05.

Based on 1,645 children randomised to the COAST trial who had a mean (standard deviation) baseline MUAC of 13.7 (1.94), and assuming 80% correlation between baseline and 90-day MUAC, we can expect change in MUAC scores at 90 days to have a standard deviation of 1.26. Whilst we don't have any other data on nutritional recovery in non-malnourished children, this is consistent with a recent trial publication ^19^ in children with severe malnutrition (including Kilifi and Coast General Hospital, Kenya) where at 90 days, a mean change in MUAC of 1.6cm (standard deviation: 1.1) was recorded.

We used simulations (with 10,000 simulated datasets) to calculate the sample size required to achieve 90% power. Assuming 5% loss to follow-up over both arms, and 5% mortality by 90 days in the control arm, a total of 840 patients would be sufficient to detect an absolute increase in MUAC change of 0.3cm together with an absolute decrease in mortality of 1%. The same sample size also provides more than 90% power to detect a larger difference in MUAC of 0.4cm or more together with no effect on mortality, or more than 80% power to detect a smaller difference in MUAC of 0.2cm together with an absolute decrease in mortality of at least 3.5%.

### Study methods and procedures

Eligible children will be identified by a nurse and clinician on duty and will be registered in the eligibility screening log. The screening form will record all patients who meet the full eligibility criteria and reasons for non-inclusion. Children enrolled into the study will have a baseline clinical assessment and laboratory investigations. The study team will work with the hospital clinicians who will be responsible for guiding management for the child according to WHO guidelines. Randomisation to nutritional strategies will start 48 hours after hospital admission.

### Randomisation procedure

Randomisation lists will be generated by the statistician at Intensive Care National Audit & Research Centre (ICNARC), London and sent to the Clinical Trials Facility, KWTRP, Kilifi, Kenya. An independent member will prepare trial randomisation envelopes before the trial, using these lists. The envelopes will be opaque and sealed and will contain a card with allocation. Children will be randomised (1:1) to receive either supplementary RUTF or not. The cards will be numbered consecutively and opened in numerical order. Clinicians will be aware of the treatment-group dose assignments, but the laboratory tests are to be performed in a blinded manner.

### Consent process

Once eligibility has been confirmed, authorised trial staff will approach parents/guardians to invite their child to take part in the trial. A patient information sheet will be provided to the parent/guardian in their usual language containing details of the COAST-Nutrition trial (see extended data). The sheet will be read aloud to those who are unable to read. The doctor/nurse will check that the information has been fully understood and parents/guardians will be encouraged to ask questions they may have about their child’s participation in the trial. This will be completed on the first day of admission, to allow baseline data to be collected. Where possible, prospective written informed consent will be sought from parents/guardians who will then be asked to sign the consent form (extended data). If parents/guardians are unable to sign, a thumbprint will be taken in lieu of a signature. A copy of the consent form will be given to the parent/guardian, the original placed in the patient’s medical notes, and a copy kept in the Investigator Site File.

### Treatment allocation

At 48 hours, following admission to hospital, eligible children will be randomised to either:

i. Supplementary feeding for 56-days (8 weeks) using one 92 g sachet (500 Kcal) per day for children under 5 years or 2 sachets for children above 5 years (1000 Kcal) of ready to use therapeutic feed (RUTF) in addition to their usual diet (intervention) or
ii. Usual diet alone (control, standard of care)

**Figure 1.**
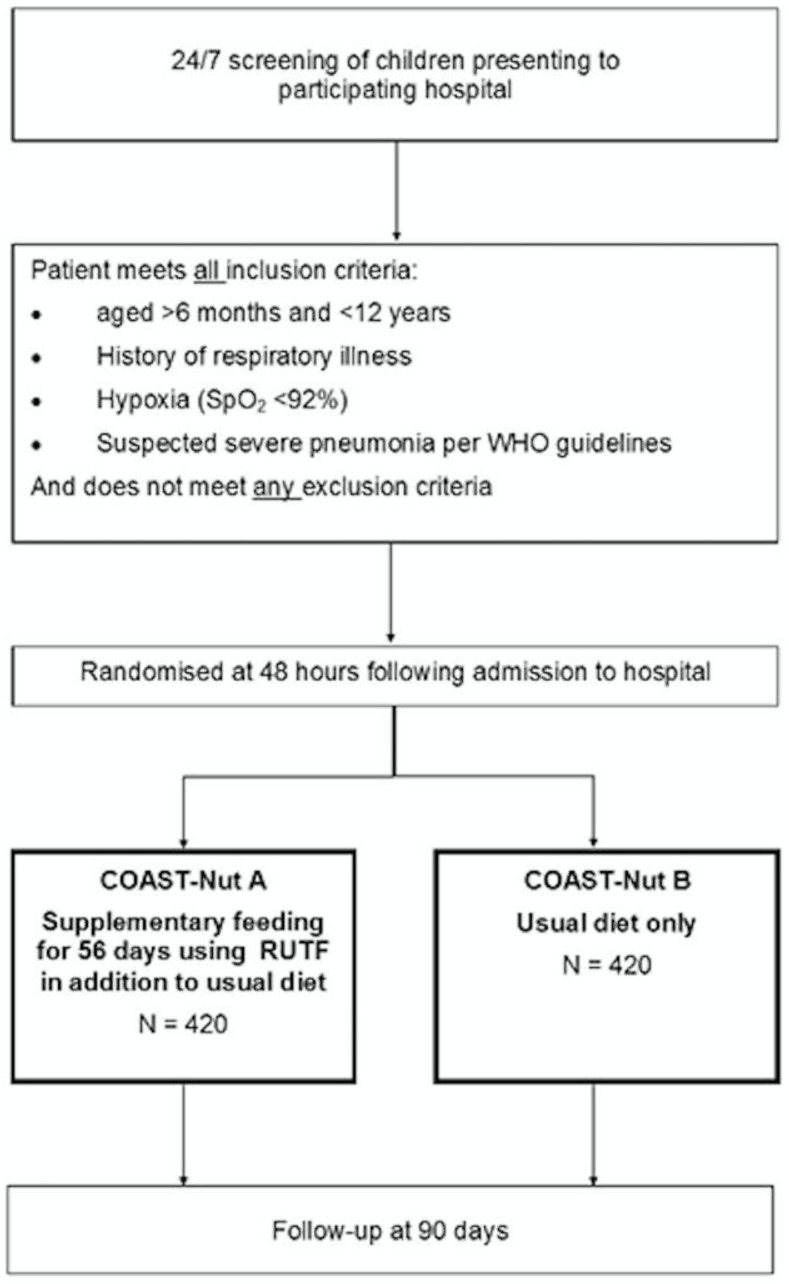
Trial flow. WHO=World Health Organization; RUTF=ready-to-use therapeutic feeds.

In stable children who are randomised to receive RUTF they will commence this after randomisation at 48-hours in addition to their dietary intake. Children who are unable to tolerate oral feeds at 48-hours will receive milk-based feeds via nasogastric tube until they are able to tolerate oral feeding. We recommend that the RUTF supplement is given with the first feed of the day. If the child is unable to complete the sachet the remainder is to be given with the following feed. Supplemental RUTF will be supplied to the child during and following hospital to day-56 post initiation. Children will be followed up to day 180 (see study flow in Figure 1).

### Trial assessment schedule

At admission clinicians will record baseline clinical assessment onto structured case report forms (CRF). During admission additional monitoring and clinical and adverse events will be captured using the case report forms (extended data). The clinical assessment and laboratory assessment schedule is captured in Table 1.

**Table 1.** Trial assessment schedule.

TIMEPOINT	*Day of admission*	*48 hours*	*Hospital discharge*	*Day 28*	*Day 90*	*Day 180*
ENROLMENT:						
Eligibility screening	X					
Consent	X					
Randomisation	x	X				
ASSESSMENTS:						
*Admission data*	X	X				
*Laboratory tests*	X			X		X
Anthropometry *	X	X	X	X	X	X
*Hospital stay data*		X	X			
*Primary outcome*					X	
*Secondary outcomes*		X	X	X	X	X
*Cost-effectiveness outcomes*			X	X		

*Including triceps skinfold thickness (TSF)

At admission the following tests will be conducted: full blood count, urea and electrolytes, point of care lactate and glucose, malaria blood slide and malaria rapid diagnostic test, blood culture. The following samples will be taken and stored for future analysis: white cell and red cell pellet (for subsequent DNA extraction for haemoglobinopathy assessment and molecular pathogen diagnosis) and nasal/oropharyngeal swabs and saliva specimen (for viral pathogen diagnosis)

Chest x-rays will also be taken when the patient is stable. In accordance with national guidelines, human immunodeficiency virus (HIV) testing will be performed after admission procedures are complete and assent given by parents or guardians. Pre- and post-test counselling will be done in accordance with routine practice.

### Health economic assessment

We will also collect data on healthcare-related costs for the trial participants, starting at randomisation and continuing for the duration of follow-up. These include

• Costs incurred by the participants and their families (e.g. transport, indirect and companion person’s costs)
• Information on hospitalisation (e.g. number, reason, and duration of stay)
• Data on other healthcare resource utilisation (e.g. outpatient visits, medications, and procedures)

### Clinical management and monitoring

During hospital admission all trial participants will receive standard of care including antibiotics (intravenous or oral), oxygen provided by mask or nasal canulae (for those with an oxygen saturations of <92%) based on WHO syndromic patient management ^17^. Children with positive malaria rapid diagnostic test (RDT) will be prescribed antimalarials (intravenous or oral). All other care will be determined by the clinical team primarily responsible for the participant’s care. Children will be reviewed twice daily thereafter until discharged from hospital. At hospital discharge we will record final diagnosis, discharge date and time. The clinical coordinator is responsible for ensuring the discharge will document and report any serious adverse events (SAEs), treatments given and non-routine treatments or investigations will also be reported.

Trial details such as MUAC at discharge and number of RUTF packets issued will also be recorded. Nutritional counselling will be done to make parents/carer understand the importance of ensuring the participant randomised to RUTF are given to improve adherence as prescribed and those that are randomised to a normal diet are given a balanced diet. The dose (number of sachets/day) will be fixed for the period of 56 days and discontinuation will only be recommended if the child is unable to tolerate the feed. Since RUTF has been used widely in African children, it has a very good safety record so we did not anticipate many children to discontinue the supplement ^20^. 

### Follow up visits

Children will be seen at 28 days, day 90 and day 180 after the day of randomisation. Transport costs after discharge and for the follow-up visit will be reimbursed to parents/guardians. At follow up the clinician will complete a symptom checklist and targeted physical examination including anthropometry. A medical history since last visit will enquire about hospital re-admissions (to be reported as a SAE) and conducted a neurocognitive function assessment (using the Developmental Milestones Checklist and an adaptation of the Kilifi Developmental Inventory ^21, 22^). Any participant lost to follow-up before 6 months will be traced for vital status (using locator data and mobile telephone contacts taken prior to discharge).

### Trial product and storage

Ready-to-use therapeutic food (RUTF) paste is a high-energy fortified food used for the treatment of SAM. It is peanut-based paste, with added sugar, vegetable oil, skimmed milk powder, and added minerals and vitamins. The RUTF for the trial, donated by UNICEF in Kenya (Plumpy’nut ^®^) is supplied by Nutriset (France). In Uganda the alternative source of RUTF, that is provided through the national nutrition programme is RATUFA, locally manufactured by Reco Limited, Kampala, Uganda. For both feeds the 92 g sachets provide 500 kcal per sachet which has a nutritional value equal to that F-100 therapeutic milk, which is recommended for nutritional rehabilitation of children with SAM. The feed can be taken direct from the sachet, as it needs no preparation or dilution prior to use. It should be stored, ideally at less than 30 ^o^C and it can be used for up to 24 months after the date of manufacture. With respect to quality control the RUTF paste should have a pleasing sweet, fresh flavour, free from foreign odours and flavours such as, but not limited to burnt, scorched, rancid, malted, sour, or stale. RUTF paste should have cream to light or orange brown colour. The RUTF paste should not have a dull, grey tinge, or another abnormal cast. It should show no evidence of excessive heating (materially darkened or scorched).

## Ancillary Studies

### Economic and cost economic analysis

The economic evaluation will be conducted from the health services perspective. This will include the costs of medication, laboratory tests as well as the ‘hotel’ costs of hospitalisation. 

The cost-effectiveness of the nutritional supplementation will compare clinical outcomes and relative costs for participants compared to those not receiving supplementation (control). The trial dataset, and other published on datasets in other trials of acutely sick children will estimate resource utilisation and unit cost data (e.g. basic costs, literature and other health-economic data) to support the cost economic analysis.

### Molecular pathogen diagnostics

The main aim of this study is to identify the role of bacteria in the aetiology of lethal pneumonia in African children. The detailed methods have been previously reported ^23^ Briefly, 16S ribosomal deoxyribonucleic acid (16S rDNA), common to all species of bacteria, will detected with using a broad-range polymerase chain reaction (PCR) and specific quantitative PCR (qPCR) will quantify the 16S rDNA subunit to directly measure the number of bacteria. We will select specific primers to ensure that qPCR is less vulnerable to background contaminants increase sensitivity. White and red cell pellets taken at enrolment into a 2ml EDTA bottle and stored at -80°C, prior to shipping to the molecular diagnostics laboratory in Kilifi where will perfom batched assays of standard 16S rDNA PCR together with a panel of 10 qPCR reactions (that target Enterobacteriaceae, a panel of anaerobes, *Streptococcus pneumoniae*, *Staphylococcus aureus* and group A streptococcus). We aim to compare the range of pathogens identified in cases (deaths) and controls (survivors) frequency matched by age group, study site and season.

### Respiratory viral diagnostics

Respiratory viruses (RV) are a major cause of acute lower respiratory tract infections worldwide ^24^, although less is known about RSV’s role in severe life-threatening disease and mortality associated with pneumonia ^25^. Since the trial includes deferred consent samples can be obtained from the sickest children, who at high mortality risk, thus provides an opportunity to elucidate the role of RSV in severe life threatening pneumonia and mortality.

Ribonucleic acid (RNA), extracted from nasal/oropharyngeal swabs taken at admission will be screened for a broad range of RVs, including influenza viruses, Respiratory Syncytial Virus (RSV), coronaviruses and rhinoviruses by multiplex real time PCR assay system ^26^. Partial and/or whole genome sequencing (WGS) of the virus positive samples, including RSV ^27^ and influenza ^28^,

From these data we will estimate prevalence and seasonal patterns respiratory viruses (RV) in this high- risk group and estimate of the risk of death in RV positive relative to negative children;

### Host genetics

Children with sickle cell disease (SCD) are at higher risk than children without SCD of hospitalisation with severe and very severe pneumonia ^29^. We propose that sickle cell status is may determine study outcomes. At the end of the trial we will batch process, using PCR, all white and red cell pellets shipped in Kilifi to describe the distribution of homozygous Sickle Cell Disease (Haemoglobin HbSS) and compare trial outcomes in children with SCD, sickle cell trait (HbAS) and normal genotype

### Anthropometry sub-study

Malnutrition and undernutrition are associated with a major decrease of muscle mass, which is decreased more than other tissues in relation to body weight. This may also have an effect on the capacity of the child to ventilate adequately in case of hypoxia ^16^. A specific effect of a reduced muscle mass on mortality has been suggested by a study in adult patients with chronic obstructive pulmonary disease showing that arm muscle area is more closely related to outcome than total lean body mass ^30^. The importance of this possible contribution of reduced muscle mass as a factor determining outcome has not been properly assessed in children suffering from pneumonia. 

**Figure 2.**
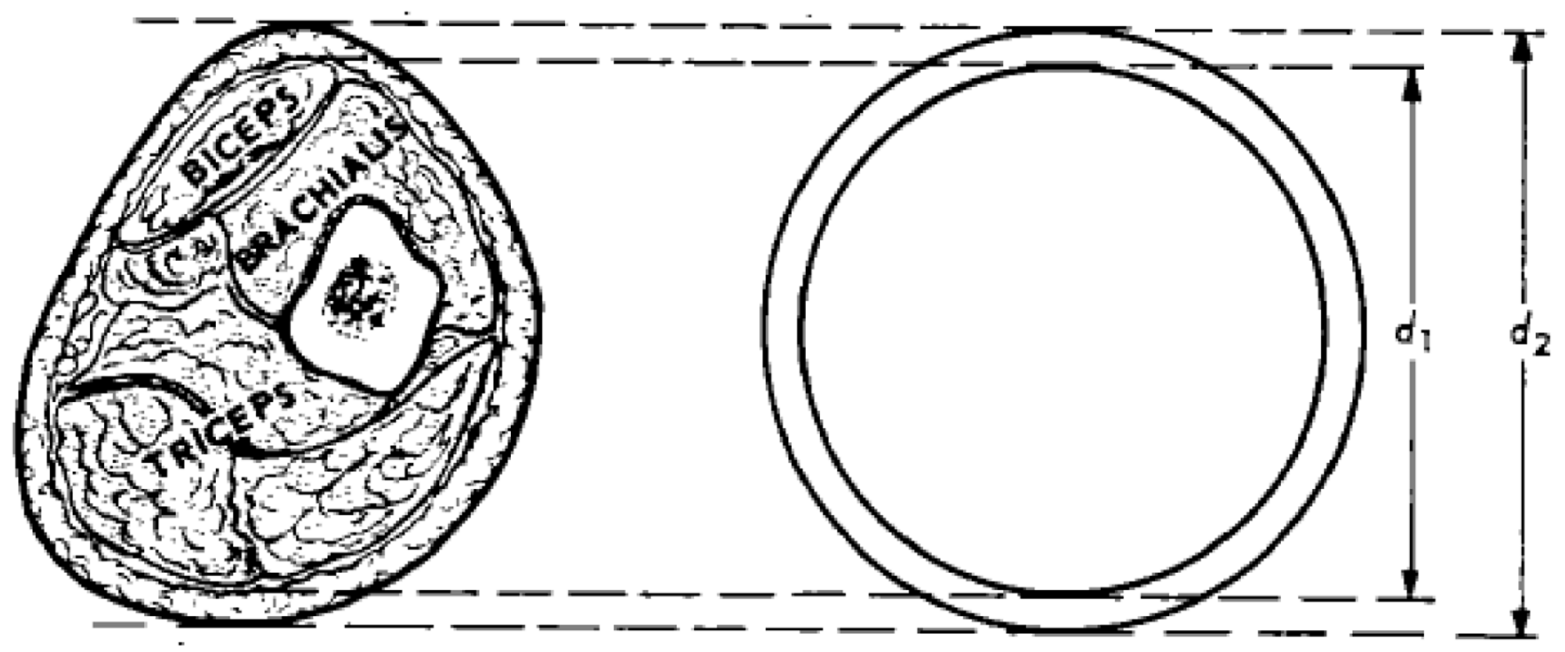
Schematic cross section upper arm. Muscle circumference (MC) can be estimated from MUAC and skinfold thickness (SF) by the formula: MC = MUAC – π SF (Jelliffe 1969) ^31^. The muscle arm area (MA) can be calculated with the formula (Rolland-Cachera *et al.*, 1997) ^32^. MA = (MUAC)²/4π – MUAC × SF/2.

We aim to assess the role of muscle mass as a factor influencing recovery of children with pneumonia. All children will have their mid-upper arm circumference (MUAC) and triceps skinfold thickness (TSF) measured at enrolment into the study as part of their initial assessment as well as on follow-up appointments i.e. discharge, day 28, 90 and 180. Arm muscle circumference (AMC) and arm muscle area (AMA) and arm fat area (AFA) will be calculated as previously described ^31, 32^ ( Figure 2). These variables will be used as proxy of muscle and fat masses. In addition to these measurements, we will measure participants’ bio impedance analysis (BIA) using the Bodystat Quadscan 4000 multi-frequency BIA Technology machine. BIA a simple non-invasive method which determines fat-free mass (FFM) or lean body weight and total body. Weight for height (WFH) and serum creatinine (a possible proxy indicator for muscle mass) and cystatin-c (to control for acute changes in renal function due to dehydration or shock) will also be part of the initial assessment. The analysis will be done on the whole sample, independently of randomisation group.

Receiver operating characteristic (ROC) curves of MUAC, AMC, AMA, AFA, WFH, serum creatinine on admission will be examined with death and treatment failure after 48h of treatment as outcome. The area under the curve (AUC) will be compared for all these ROC curves.

### Putative biomarkers of pneumonia

Since WHO clinical criteria for identification of pneumonia are very broad, prioritising sensitivity over specificity ^33^, this results in the over diagnosis of the clinical syndrome of pneumonia, particularly in sub-Saharan Africa ^34^. In order to inform a policy of targeting treatments to those who will receive the most benefit this sub-study aims to establish whether incorporating within the COAST-Nutrition trial an evaluation of previously validated point-of-care tests and other biomarkers refines the diagnosis of pneumonia in children.

All children in the trial have, following consent plasma stored at admission and a chest radiograph. We will conduct the following assays: c-reactive protein, procalcitonin; neutrophil gelatinase-associated lipocalin previously been shown to be more specific for bacterial infection and pneumonia ^35– 38^ and any new novel biomarker identified through a literature search. 

A dedicated clinician will be trained to review the chest x-rays and report them according to WHO standardised interpretation of chest radiographs. The WHO methodology has since been adopted by many studies of vaccine efficacy ^39^ and descriptive epidemiology of pneumonia cases ^40^ and with training has good inter observer agreement for the detection of ‘any consolidation’ in other pneumonia studies ^41^ and evaluations of the WHO methodology. We will use WHO standardised methodology for interpretation of paediatric chest X-ray, which will be evaluated by 2 independent observers (one radiologist and the clinician/research fellow). All interpretations of x-rays and laboratory assays will be done blind to the child’s clinical diagnosis and randomisation strategy.

A retrospective analysis will investigate whether

1. Point of care tests and/or biomarkers alone or in combination with clinical signs can classify individual into groups with defined characteristics e.g. radiographic consolidation to assist with refining the primary and secondary endpoints for the trial.
2. Point of care tests and biomarkers alone or in combination with clinical signs in children with and without radiographic pneumonia correlate reliably with bacterial and viral pathogens identified in the relevant ancillary studies (see above)
3. whether biomarker prediction can refine case definitions and linking these to the primary and secondary outcome of the interventions in the trial (ie stratify risk/benefit and/or identify specific subgroups).

## Trial outcome measures

### Primary outcomes

Primary outcome is change in mid-upper arm circumference (MUAC) at 90 days as a composite with 90-day mortality.

### Secondary outcomes

The key secondary outcome measures are: 

•    Survival to 28 days and 180 days (6 months) 

•    Disability-free survival to 28 days 

•    Re-admission to hospital by 28 and day 180 days 

•    Neurocognitive sequelae at 90 days 

•    Anthropometric status by 28 days, day 90 and day 180 (MUAC, TST and WHZ) 

•    Adverse events associated with RUTF 

## Trial monitoring

### Data monitoring committee

An independent DMC (see composition at the end of the protocol) will meet to review unblinded data after randomisation of 420 children. They will review enrolment, safety, adherence to the trial protocol and efficacy data in strict confidence. Their terms are covered in the DMC charter, signed by the chair and trial statistician. The DMC is comprised of a chair, an independent statistician and two other independent members. None declared conflicts of interest. The DMC will provide advice on the conduct of the trial to the Trial Steering Committee (TSC). Guidelines to recommend early termination will be based on a Peto-Haybittle stopping rule (P<0.001). A recommendation to discontinue recruitment, in all participants or in selected subgroups, will be made only if the results are likely to convince the general clinical community and participants in the COAST-Nutrition trial. 

### Onsite monitoring

The main coordinating centre for trial monitoring will be the Clinical Trial Facility in KWTP, Kilifi. Monitors independent from the trial will set out in a monitoring management Plan that will determine the frequency of visits and the degree of source document verification against the case record forms. Monitors will attend the site initiation visits (SIV) and observe trial procedures training, and oversee reporting guidelines for adverse events of study interventions Trial teams are required to have formal GCP training which incorporate specific operation guidance for the COAST Nutrition trial. On-site monitoring visits will occur annuallly. Details of the scope of the monitoring visits have previously been reported ^23^.

### Data management

All clinical and laboratory data will be recorded in the CRF and stored with a unique serial number identifier. Following initial verification against source documents by the study site coordinator the case report forms will be entered (double data entry) onto OpenClinica Version 3. The databases are regularly backed up, with copies stored both on and off site. The paper records (dedicated source documents and CRFs) will be archived in locked cabinets, which have limited access with prior authorisation at the respective study sites by the Principal investigator. The data management plan, CRF and information and consent form can be found as extended data ^42^.

Methods for data collection and data entry will be documented in trial Standard Operating Procedures. Technical details of variables and their coding will be recorded in a detailed Dataset Specification. Data will be checked centrally by KCTF which will validation checks for completeness, accuracy and consistency. Discrepancies and missing data queries will be sent from KCTF back to the trial sites. The study site coordinator is responsible for ensuring that queries and inconsistencies are resolved in a timely manner, and includes correction of relevant paper CRFs and the re-entry onto the trial database. An audit log will keep track of all changes made to the database. The system will be maintained and hosted by the Kilifi Clinical Trials Facility (KCTF)

All data will be partially anonymised and study participants can only be identified by a unique patient identification number. In compliance with GCP requirements no patient identifiable information will be recorded on the trial database. Prior to data extract the data will be examined for inconsistencies by the data manager and statistician which will be and fed back to study sites for corrections.

### Confidentiality

Participants’ identification data will be required for the registration process. The KCTF and ICNARC CTU will preserve the confidentiality of participants taking part in the study, in compliance data protection requirements, in the respective countries where the research is being conducted. Prior to presentation or publication of any result data will be anonymised before data analysis. 

### Data sharing

After completion of the study, requests for data access from researchers outside the study team will be considered by the trial management team and clinical trials unit. We will ensure these do not conflict with ongoing analyses and PhD student projects. When indicated the requestors will be asked to develop scientific protocols for approval of secondary analyses. The potential to share data to external bodies is included in the participant information and consent form.

### Statistical analysis

The analyses will be described in detail in a full statistical analysis plan. This section summarises the main issues. Stata version 16.1 will be used to analyse the data. The primary outcome (change in MUAC base line as a composite with mortality to day 90) will be analysed using rank-based methods (Mann-Whitney test). The individual components of the composite endpoint (change in MUAC in patients surviving to day 90, and mortality by day 90) will be additionally reported by arm, and compared using both unadjusted (unpaired t-test and log-rank test) and adjusted methods (generalised linear models and Cox regression, adjusting for treatment allocation and trial site)

Secondary outcomes will be analysed using generalised linear models, (with the same model structure as for the primary outcome above, and corrected for a limited number of pre-specified clinically important admission variables), as follows:

• Re-admission to hospital by 28 days, neurocognitive sequelae at 28 days and disability-free survival to 28 days and day 180 will be analysed as binary outcomes using logistic regression, with all deaths included in the failure group.
• Length of initial hospital stay and anthropometric status will be analysed as continuous outcomes using linear regression.
• Survival to 28 and 180 days will be analysed as a time-to-event outcome using a Cox proportional hazards model.
• Resolution of neurocognitive sequelae at 90 days will be analysed as a binary outcome using logistic regression, only among those with neurocognitive sequelae at 28 days.

Additional hypothesis-generating analyses will investigate whether there is any evidence for a different impact of the interventions according to the following categorical variables: fever; malaria; microbiological evidence of sepsis (blood culture or retrospective molecular diagnosis); radiographic evidence of pneumonia; HIV; severe anaemia (haemoglobin <5g/dl); and undiagnosed sickle cell disease. Subgroup analyses will be conducted by testing the significance of interaction terms in the regression models as specified above.

### Ethics statement

Ethical approval has been obtained from Imperial College London Research Ethics Committee (15IC3100); School of Medicine Makerere University REC (2016-030 and the amendment 2020-155) in Uganda and KEMRI Scientific and Ethics Review Unit KEMRI/SERU/CGMRC-C/0053/3300 and amendment. C 215/4109 in Kenya. The trial will be conducted in accordance with the recommendations for research on human subjects in the Declaration of Helsinki ^43^, the ICH-GCP guidelines (E6(R1), 1996) and the applicable national regulations.

The trial was registered on ISRCTN (ISRCTN10829073, 6 ^th^ June 2018) and on the Pan African Clinical Trials Registry (PACTR202106635355751, 2 ^nd^ June 2021).

### Safety

The randomised trial will be conducted in children who are most likely to benefit from the treatment. Children with SAM will not be enrolled in the trial as they will all receive full nutritional support. Any child developing SAM during follow up will be referred to the nutritional services for treatment. Infants under 6 months are excluded as they should be exclusively breast fed. We will minimise the risks of cannula insertion and phlebotomy by pretrial training in phlebotomy technique and regular cannula site inspection which is included in a standard operating procedure. Less than 1ml/kg of blood will be drawn for research purposes at any one time.

### Benefits

Pretrial training of the dedicated study team will include specific training on general management of severe malaria and its complications. A manual of operations will provide clear management guidelines as well as the details of trial conduct and procedures. Children enrolled in the trial will therefore receive a higher quality of care than those managed routinely. Following discharge the parents/children will be encouraged to keep in touch with the clinical coordinator and if their child is ill to return to clinic/hospital so they can be assessed and treated (or admitted to hospital) by the COAST-Nutrition team.

## Plans for dissemination of the study outcomes

### Public engagement

Results from this trial will be disseminated locally through community meetings and national meetings with the wider healthcare professional community. These systems have been developed for dissemination of MRC FEAST ^44^ and TRACT ^45^ trial results and will be adapted to dissemination for the COAST-Nutrition trial.

### National and international policymakers

The principal investigators for the COAST-Nutrition trial have discussed the study with Ministry of Health staff and nutritional services. When the results are available, we will provide a summary briefing highlighting the trial results in different and what then next steps will be. Whilst the current study will go some way towards addressing whether nutritional support could be used as supportive therapy following an admission with severe pneumonia future trials should consider pragmatic designs to ensure results are applicable to health services in Africa.

## Discussion

Severe pneumonia remains is a key cause of paediatric admission in many countries in Africa. Both short term and long-term outcomes are poor. Early and supplementary feeding to avert post-discharge mortality in those surviving hospital admission has been recommended but not yet tested in clinical trials. However, there is no specific guidance given on what type of nutritional support should be given and for how long.

In 2017 the investigator group initiated the Children’s Oxygen Administration Strategies Trial (COAST) funded by MRC/DFID/ Wellcome Trust’s Joint Global Health Trials which was designed to simultaneously evaluate two related oxygenation strategies in 4200 children to reduce short-term mortality at 48-hours (primary endpoint) and longer-term morbidity and mortality to 28 days in a large Phase III trial with a factorial design ^46^. In 2017 the trial group received co-funding from the European and Developing Countries Clinical Trials Partnership (EDCTP) to maximise the impact of the trial infrastructure using the platform to enroll children into a further trial (COAST-Nutrition) at 48-hours examining whether supplemented feeding to Day-56 improves 90-day survival. Additional objectives of the study included evaluation whether point-of-care tests and other biomarkers could predict clinical and radiologically-defined pneumonia in those with viral and bacterial aetiologies in order to refine endpoints for the COAST trial and future targeting of definitive and supportive therapies (including tailoring antibiotic management in hospitals lacking microbiology services). Thus, the objectives the COAST and COAST-Nutrition trials were designed to evaluate two major barriers to improving in-patient and post-discharge mortality. This was by evaluating in a pragmatic randomised controlled trial (RCT) two linked components of oxygen delivery (which could form an integrated treatment package for oxygen administration in hospitals with no access to mechanical ventilation) and improved nutritional support post-discharge to improve long-term outcome. An integrated economic evaluation was included both to identify whether the additional costs of each of the interventions are proportionate to the health benefits and to inform future widespread implementation in terms of value for money. A major reason for including both questions into one RCT is efficiency - each targeting a different mechanism for reducing mortality and morbidity and it is more economical to run one large RCT, COAST, than two separate RCTs. At the time the trial was designed and started we searched ISRCTN and Trials.gov and found that there were currently no randomised controlled trials (RCT) assessing either the targeted use of oxygen therapy (including threshold for giving oxygen) in children or assessing nutritional support in children hospitalised with putative pneumonia.

The start of recruitment into the COAST Nutrition trial in Kenya was on 12 ^th^ August 2018 and in Uganda on date 28 ^th^ November 2018. Enrolment into the trial was slow owing to repeated stoppages of the COAST trial in Uganda. After the 5 ^th^ stoppage the TSC recommended the COAST oxygen trial to terminate, based on feasibility but recommended the COAST Nutrition trial to continue once the protocol had been amended (see relevant sections below). The results of the COAST oxygen trial have now been published as well as an editorial authored by members of the TSC ^47, 48^.

## Trial status

Trial enrolment restarted in November 2020 in Ugandan sites, after a long break due to the stoppage of COAST main study by the TSC in February 2020 and the interruption of research caused by the coronavirus disease 2019 (COVID-19) pandemic. We had to submit the study as a new proposal to School of Medicine Research Ethics Committee (SOMREC) and Uganda National Council of Science and Technology (UNCST) and were finally granted approval on 8 ^th^ September 2020 and 10 ^th^ October 2020, respectively. We also submitted the revised protocol to the Kenyan ethics and regulatory authority and were granted approval on 20 ^th^ November 2020 and 29 ^th^ March 2021. Coupled with challenges due to the ongoing COVID-19 pandemic the recruitment has been significantly slow. To date 420 children have been enrolled into the COAST-Nutrition trial. 

## Protocol version changes

The original protocol was submitted as an oxygen strategies trial ^46^. In 2017 we received additional funding from EDCTP to add in a second randomisation at 48 hours to the nutritional intervention COAST Nutrition. The protocol was therefore amended, amendment version 3.0 dated 21/08/2017 to include COAST Nutrition and was granted full approval by ICREC on 27 ^th^ September 2017, further minor revisions which were further approved by local regulatory authorities. Following the TSC recommendation in 2020 to terminate the COAST oxygen trial the protocol was amended to remove justifications for the oxygen trial and its randomisation. Preliminary approval was given by ICREC pending national ERC approval. These were received in Uganda on 28 ^th^ August 2020 (country-specific addendum called Nutrition Therapy in Pneumonia; NuTiP), on 7 ^th^ October 2020 in Kenya and full approval by ICREC on 22 ^nd^ April 2021 (and protocol version renamed version 4). 

**Table:** 

Version no.	Date	Amendment no.	Protocol Section (no./title)
v1.0	25 ^th^ January 2016	N/A	COAST protocol
v2.0	7 ^th^ July 2016	1st	Blood gases dropped
v2.1	11 ^th^ January 2017	2nd	One investigator change
v3.0	14 ^th^ August 2017	3rd	COAST nutrition details included
V4.0	17 ^th^ February 2020	4th	COAST Nutrition only; oxygen strategies dropped

## Roles and responsibilities

### Role of study sponsor and funders

The sponsor and funder played no role in in study design and will play no role in data collection, trial management, analysis and interpretation of data and manuscript preparation the decision to submit the report for publication.

## Trial management group

Kathryn Maitland, Hellen Mnjalla, Ayub Mpoya, Christabel Mogaka, Phyles Maitha, Dennis Aromut and Sarah Kiguli. 

## Trial steering committee

Professor Elizabeth Molyneux, OBE (Chairman): College of Medicine, Blantyre Malawi; Dr Jane Crawley: University of Oxford; Dr Irene Lubega: Department of Paediatrics Makerere University College of Health Sciences, Uganda, Prof Mark Peters, Great Ormond Street Hospital and University College London, UK. 

## Data monitoring committee

Prof. Timothy Peto (Chair): University of Oxford; Dr Jim Todd (Trial Statistician): London School of Hygiene and Tropical Medicine; Professor Philippa Musoke: Makerere University College of Health Sciences, Uganda and Prof Calum Semple (OBE), Child Health and Outbreak Medicine at the University of Liverpool, UK

## Sponsor

Imperial College London is the main research Sponsor for this study. For further information regarding the sponsorship conditions, please contact the Head of Regulatory Compliance at:

Joint Research Office, Room 221b, Medical School Building, St Mary’s Campus, Norfolk Place, London, W2 1PG. Telephone: +44 (0) 020 7594 1872.

## Data availability

### Underlying data

No underlying data are associated with this article.

### Extended data

Imperial College Research Data Repository: COAST-Nutrition Extended Data.

https://doi.org/10.14469/hpc/8370 ^42^.

This project contains the following extended data:

- COAST_N Merged CRF's April 2021 V 1.0(latest)-Kilifi.pdf
- COAST Nutrition v 1.1Final- ICF_English-Clean.pdf
- Appendix 3 Data Management Plan COAST Nutrition.pdf

### Reporting guidelines

Imperial College Research Data Repository: SPIRIT checklist for ‘Children’s Oxygen Administration Strategies And Nutrition Trial (COAST-Nutrition)’. https://doi.og/10.14469/hpc/8369 ^15^.

Data are available under the terms of the Creative Commons Zero "No rights reserved" data waiver (CC0 1.0 Public domain dedication).

## References

[1] Black RE Cousens S Johnson HL : Global, regional, and national causes of child mortality in 2008: a systematic analysis. *Lancet.* 2010;375(9730):1969–1987. 10.1016/S0140-6736(10)60549-1 20466419

[2] Mwaniki MK Nokes DJ Ignas J : Emergency triage assessment for hypoxaemia in neonates and young children in a Kenyan hospital: an observational study. *Bull World Health Organ.* 2009;87(4):263–270. 10.2471/blt.07.049148 19551234 PMC2672576

[3] Banajeh SM : Outcome for children under 5 years hospitalized with severe acute lower respiratory tract infections in Yemen: a 5 year experience. *J Trop Pediatr.* 1998;44(6):343–346. 10.1093/tropej/44.6.343 9972077

[4] Qazi S Aboubaker S MacLean R : Ending preventable child deaths from pneumonia and diarrhoea by 2025. Development of the integrated Global Action Plan for the Prevention and Control of Pneumonia and Diarrhoea. *Arch Dis Child.* 2015;100 Suppl 1:S23–28. 10.1136/archdischild-2013-305429 25613963

[5] Baudouin SV Evans TW : Nutritional support in critical care. *Clin Chest Med.* 2003;24(4):633–644. 10.1016/s0272-5231(03)00101-1 14710695

[6] Debaveye Y Van den Berghe G : Risks and benefits of nutritional support during critical illness. *Annu Rev Nutr.* 2006;26:513–538. 10.1146/annurev.nutr.26.061505.111307 16848718

[7] Moïsi JC Gatakaa H Berkley JA : Excess child mortality after discharge from hospital in Kilifi, Kenya: a retrospective cohort analysis. *Bull World Health Organ.* 2011;89(10):725–732,732A. 10.2471/BLT.11.089235 22084510 PMC3209982

[8] Ngari MM Fegan G Mwangome MK : Mortality after Inpatient Treatment for Severe Pneumonia in Children: a Cohort Study. *Paediatr Perinat Epidemiol.* 2017;31(3):233–242. 10.1111/ppe.12348 28317139 PMC5434848

[9] Ziegler TR Gatzen C Wilmore DW : Strategies for attenuating protein-catabolic responses in the critically ill. *Annu Rev Med.* 1994;45:459–480. 10.1146/annurev.med.45.1.459 8198396

[10] Shoham J Duffield A : Proceedings of the World Health Organization/ UNICEF/World Food Programme/United Nations High Commissioner for Refugees Consultation on the management of moderate malnutrition in children under 5 years of age. *Food Nutr Bull.* 2009;30(3 Suppl):S464–474. 10.1177/15648265090303S306 19998867

[11] Golden MH : Proposed recommended nutrient densities for moderately malnourished children. *Food Nutr Bull.* 2009;30(3 Suppl):S267–342. 10.1177/15648265090303S302 19998863

[12] Goossens S Bekele Y Yun O : Mid-upper arm circumference based nutrition programming: evidence for a new approach in regions with high burden of acute malnutrition. *PLoS One.* 2012;7(11):e49320. 10.1371/journal.pone.0049320 23189140 PMC3506602

[13] Maust A Koroma AS Abla C : Severe and Moderate Acute Malnutrition Can Be Successfully Managed with an Integrated Protocol in Sierra Leone. *J Nutr.* 2015;145(11):2604–2609. 10.3945/jn.115.214957 26423737

[14] Bailey J Chase R Kerac M : Combined protocol for SAM/MAM treatment: The ComPAS study.In. Edited by Exchange F;2016. Reference Source

[15] Maitland K : COAST-Nutrition Extended Data.Version 1. Imperial College London Data Repository. Dataset.2021. 10.14469/hpc/8370

[16] Briend A Khara T Dolan C : Wasting and stunting--similarities and differences: policy and programmatic implications. *Food Nutr Bull.* 2015;36(1 Suppl):S15–23. 10.1177/15648265150361S103 25902610

[17] WHO Guidelines Approved by the Guidelines Review Committee: Pocket Book of Hospital Care for Children: Guidelines for the management of common childhood illnesses.In., Second edn. Geneva: World Health Organization;2013. 24006557

[18] Bejon P Mohammed S Mwangi I : Fraction of all hospital admissions and deaths attributable to malnutrition among children in rural Kenya. *Am J Clin Nutr.* 2008;88(6):1626–1631. 10.3945/ajcn.2008.26510 19064524 PMC2635111

[19] Berkley JA Ngari M Thitiri J : Daily co-trimoxazole prophylaxis to prevent mortality in children with complicated severe acute malnutrition: a multicentre, double-blind, randomised placebo-controlled trial. *Lancet Glob Health.* 2016;4(7):e464–473. 10.1016/S2214-109X(16)30096-1 27265353 PMC6132285

[20] Das JK Salam RA Saeed M : Effectiveness of Interventions for Managing Acute Malnutrition in Children under Five Years of Age in Low-Income and Middle-Income Countries: A Systematic Review and Meta-Analysis. *Nutrients.* 2020;12(1):116. 10.3390/nu12010116 31906272 PMC7019612

[21] Abubakar A Holding P Van de Vijver F : Developmental monitoring using caregiver reports in a resource-limited setting: the case of Kilifi, Kenya. *Acta Paediatr.* 2010;99(2):291–297. 10.1111/j.1651-2227.2009.01561.x 20353499 PMC2814084

[22] Abubakar A Holding P van Baar A : Monitoring psychomotor development in a resource-limited setting: an evaluation of the Kilifi Developmental Inventory. *Ann Trop Paediatr.* 2008;28(3):217–226. 10.1179/146532808X335679 18727851 PMC3908377

[23] Maitland K Kiguli S Opoka RO : Children’s Oxygen Administration Strategies Trial (COAST): A randomised controlled trial of high flow versus oxygen versus control in African children with severe pneumonia [version 1; peer review: 1 approved, 1 approved with reservations]. *Wellcome Open Res.* 2017;2:100. 10.12688/wellcomeopenres.12747.1 29383331 PMC5771148

[24] Shi T McLean K Campbell H : Aetiological role of common respiratory viruses in acute lower respiratory infections in children under five years: A systematic review and meta-analysis. *J Glob Health.* 2015;5(1):010408. 10.7189/jogh.05.010408 26445672 PMC4593292

[25] Nair H Brooks WA Katz M : Global burden of respiratory infections due to seasonal influenza in young children: a systematic review and meta-analysis. *Lancet.* 2011;378(9807):1917–1930. 10.1016/S0140-6736(11)61051-9 22078723

[26] Hammitt LL Kazungu S Welch S : Added value of an oropharyngeal swab in detection of viruses in children hospitalized with lower respiratory tract infection. *J Clin Microbiol.* 2011;49(6):2318–2320. 10.1128/JCM.02605-10 21490188 PMC3122752

[27] Agoti CN Otieno JR Munywoki PK : Local evolutionary patterns of human respiratory syncytial virus derived from whole-genome sequencing. *J Virol.* 2015;89(7):3444–3454. 10.1128/JVI.03391-14 25609811 PMC4403408

[28] Baillie GJ Galiano M Agapow PM : Evolutionary dynamics of local pandemic H1N1/2009 influenza virus lineages revealed by whole-genome analysis. *J Virol.* 2012;86(1):11–18. 10.1128/JVI.05347-11 22013031 PMC3255882

[29] Uyoga S Macharia AW Mochamah G : The epidemiology of sickle cell disease in children recruited in infancy in Kilifi, Kenya: a prospective cohort study. *Lancet Glob Health.* 2019;7(10):e1458–e1466. 10.1016/S2214-109X(19)30328-6 31451441 PMC7024980

[30] Soler-Cataluna JJ Sanchez-Sanchez L Martinez-Garcia MA : Mid-arm muscle area is a better predictor of mortality than body mass index in COPD. *Chest.* 2005;128(4):2108–2115. 10.1378/chest.128.4.2108 16236862

[31] Jelliffe E Jelliffe D : The Arm Circumference as a Public Health Index of Protein-Calorie Malnutrition of Early Childhood. *J Trop Pediatr.* 1969;15:179–188. 5309233

[32] Rolland-Cachera MF Brambilla P Manzoni P : Body composition assessed on the basis of arm circumference and triceps skinfold thickness: a new index validated in children by magnetic resonance imaging. *Am J Clin Nutr.* 1997;65(6):1709–1713. 10.1093/ajcn/65.6.1709 9174464

[33] Maitland K : New diagnostics for common childhood infections. * N Engl J Med.* 2014;370(9):875–877. 10.1056/NEJMe1316036 24571761

[34] Graham SM English M Hazir T : Challenges to improving case management of childhood pneumonia at health facilities in resource-limited settings. *Bull World Health Organ.* 2008;86(5):349–355. 10.2471/blt.07.048512 18545737 PMC2647436

[35] Huang H Ideh RC Gitau E : Discovery and validation of biomarkers to guide clinical management of pneumonia in African children. *Clin Infect Dis.* 2014;58(12):1707–1715. 10.1093/cid/ciu202 24696240 PMC4036688

[36] Erdman LK D'Acremont V Hayford K : Biomarkers of Host Response Predict Primary End-Point Radiological Pneumonia in Tanzanian Children with Clinical Pneumonia: A Prospective Cohort Study. *PLoS One.* 2015;10(9):e0137592. 10.1371/journal.pone.0137592 26366571 PMC4569067

[37] Riedel S : Procalcitonin and the role of biomarkers in the diagnosis and management of sepsis. *Diagn Microbiol Infect Dis.* 2012;73(3):221–227. 10.1016/j.diagmicrobio.2012.05.002 22704255

[38] Irwin AD Marriage F Mankhambo LA : Novel biomarker combination improves the diagnosis of serious bacterial infections in Malawian children. *BMC Med Genomics.* 2012;5:13. 10.1186/1755-8794-5-13 22559298 PMC3528639

[39] Roca A Sigauque B Quinto L : Estimating the vaccine-preventable burden of hospitalized pneumonia among young Mozambican children. *Vaccine.* 2010;28(30):4851–4857. 10.1016/j.vaccine.2010.03.060 20392430

[40] Enwere G Cheung YB Zaman SM : Epidemiology and clinical features of pneumonia according to radiographic findings in Gambian children. *Trop Med Int Health.* 2007;12(11):1377–1385. 10.1111/j.1365-3156.2007.01922.x 18045264

[41] Xavier-Souza G Vilas-Boas AL Fontoura MSH : The inter-observer variation of chest radiograph reading in acute lower respiratory tract infection among children. *Pediatr Pulmonol.* 2013;48(5):464–469. 10.1002/ppul.22644 22888091

[42] Maitland K : SPIRIT Checklist: Children’s Oxygen Administration Strategies And Nutrition Trial (COAST-Nutrition): protocol for a Phase II randomised controlled trial.Version 1. Imperial College London Data Repository. Dataset.2021. 10.14469/hpc/8369

[43] Declaration of Helsinki - Ethical Principles for Medical Research Involving Human Subjects. In: Edited by Association WM. Helsinki;1964. Reference Source

[44] Maitland K Kiguli S Opoka RO : Mortality after fluid bolus in African children with severe infection. *N Engl J Med.* 2011;364(26):2483–2495. 10.1056/NEJMoa1101549 21615299

[45] Maitland K Kiguli S Olupot-Olupot P : Immediate Transfusion in African Children with Uncomplicated Severe Anemia. *N Engl J Med.* 2019;381(5):407–419. 10.1056/NEJMoa1900105 31365799 PMC7611152

[46] Maitland K Kiguli S Opoka RO : Children's Oxygen Administration Strategies Trial (COAST): A randomised controlled trial of high flow versus oxygen versus control in African children with severe pneumonia [version 2; peer review: 2 approved]. *Wellcome Open Res.* 2018;2:100. 10.12688/wellcomeopenres.12747.2 29383331 PMC5771148

[47] Maitland K Kiguli S Olupot-Olupot P : Randomised controlled trial of oxygen therapy and high-flow nasal therapy in African children with pneumonia. *Intensive Care Med.* 2021;47(5):566–576. 10.1007/s00134-021-06385-3 33954839 PMC8098782

[48] Peters MJ Macharia W Molyneux E : A COASTal view: where prior beliefs and uncertainty collide. *Intensive Care Med.* 2021;47(5):591–593. 10.1007/s00134-021-06406-1 33904949

