# Supplementary material for: Children’s Oxygen Administration Strategies And Nutrition Trial (COAST-Nutrition): a protocol for a phase II randomised controlled trial
Source: Wellcome Open Res. 2021 Oct 18;6:221. Originally published 2021 Sep 3. [Version 2] doi: 10.12688/wellcomeopenres.17123.2 (PMC8529399; doi:10.12688/wellcomeopenres.17123.2)
Supplement: Peer review history

# Peer review: Reviewer response for version 2

**DOI:** 10.21956/wellcomeopenres.19091.r46507

**Published:** 2021-10-20

**Authors:** Jamie Rylance

**Affiliations:** 1 Liverpool School of Tropical Medicine, Liverpool, UK; 2 Malawi-Liverpool-Wellcome Trust, Blantyre, Malawi

All of the issues highlighted in the first review have been addressed fully. Many thanks.

Is the study design appropriate for the research question?

Yes

Is the rationale for, and objectives of, the study clearly described?

Yes

Are sufficient details of the methods provided to allow replication by others?

Yes

Are the datasets clearly presented in a useable and accessible format?

Not applicable

Reviewer Expertise:

I am an adult general physician and clinical researcher with experience of cohort studies and RCTs in sub-Saharan Africa.

I confirm that I have read this submission and believe that I have an appropriate level of expertise to confirm that it is of an acceptable scientific standard.

# Peer review: Reviewer response for version 2

**DOI:** 10.21956/wellcomeopenres.19091.r46506

**Published:** 2021-10-18

**Authors:** Carina King

**Affiliations:** 1 Global Public Health, Karolinska Institute, Stockholm, Sweden

Thank you for all the clarifications - I am really looking forward to seeing the results of this trial!

Is the study design appropriate for the research question?

Yes

Is the rationale for, and objectives of, the study clearly described?

Yes

Are sufficient details of the methods provided to allow replication by others?

Partly

Are the datasets clearly presented in a useable and accessible format?

Not applicable

Reviewer Expertise:

Epidemiologist

I confirm that I have read this submission and believe that I have an appropriate level of expertise to confirm that it is of an acceptable scientific standard.

# Peer review: Reviewer response for version 1

**DOI:** 10.21956/wellcomeopenres.18914.r45741

**Published:** 2021-09-21

**Authors:** Jamie Rylance

**Affiliations:** 1 Liverpool School of Tropical Medicine, Liverpool, UK; 2 Malawi-Liverpool-Wellcome Trust, Blantyre, Malawi

Many thanks for asking me to review this manuscript; I hope the comments and requested clarifications are helpful.

 This article describes a phase 2 randomised controlled trial of supplemental feeding with Ready to Use Formula (RUTF) following hospital admission for severe pneumonia in children aged 6 to 12 months in Kenya and Uganda. The composite endpoint includes a summary measure of nutrition (Mid Upper Arm Circumference) and 90 day mortality. The comparator is usual nutrition (control arm). The history of the protocol is complex, having been previously combined with an RCT of oxygen support (COAST) which has now been discontinued for reasons of non-feasibility secondary to low recruitment rates. This article describes both this, and presents a definitive description of the nutrition only component (COAST-Nutrition).

 The rationale for nutrition support is supported by generally poor quality evidence, and descriptions of current practice. While the authors do note that “Early nutritional support is commonly practices in a wide variety of critically ill patients, including those with sepsis”, it would be preferable to cite evidence rather than practice. Where evidence is missing, or contradictory or confused (which is probably the case for sepsis at present), this should be more clearly stated. There are some data from a Kenyan paediatric cohort (Ngari *et al.*) used partly to justify the potential for nutritional support to alter mortality. These data do show a strong association of nutrition (and HIV) with death. However, the analysis here examined the association in a group which are *a priori* excluded from the COAST Nutrition trial (severe acute malnutrition is an exclusion criteria), and this should be made clear.

 The trial is general is well and comprehensively described. I am unsure whether the trial is truly intended to be a phase 2 trial – it contains more participants than might usually be expected for phase 2, although it does have an efficacy endpoint. Given this, it would be useful to clarify language in the manuscript. For example, “This trial… will provide preliminary evidence for health services in Africa on the basis of clinical effectiveness and costs” could be confused by the use of “effectiveness” here. Similarly, “cost-effectiveness outcomes” in Table 1 might be better reflected as by the term “cost economic analysis used elsewhere in the document”. 

**Consent:** There is some justification later in the document for deferred consent, and this seems appropriate. However, in the main “Consent process” this should be made explicit and more clearly described.

**SAE reporting:** The process for SAE reporting and decision making is not clear. It is highly likely that there will be many SAEs (mortality is high), and it is noted that the clinical coordinator is responsible for reporting, and that the data monitoring committee will review at half-way to completion. In the interim periods, are there mechanisms for review of these, and who are they reported to. Another large source of SAEs is likely to be events which cause prolongation of hospital stay – how will these be ascertained and recorded?

**Outcomes:** These are well described. The use of the Developmental Milestones Checklist needs to be specified i.e. as “no neurocognitive deficit” is a secondary outcome; how will the tool be used to generate this binary variable?

**Notification of clinical information:** Sickle cell outcomes will be determined by batch processing after completion. However, this represents important clinical information which has repercussions for clinical care. What is the mechanism for getting this information back to children and their families?

**Data sharing:** After completion of the study, the trial management team may not be coherent or properly constituted. If requests for data are channelled is this manner, what is the longer term plan to make the data accessible?

**Minor issues:**

- Definitions are not always laid out clearly. The term “very severe pneumonia” is used in the introduction – is this conflated with the IMCI “very severe disease” category of having a general danger sign? Similarly, while the WHO definition of “severe pneumonia” may be widely known, it would be helpful to restate this here.
- The recommendation for RUTF in severe acute malnutrition of 175-200kcal/kg/day should be referenced more clearly. I can find it in neither of the adjoining references, but perhaps this comes from the Pocket Book of Hospital Care for Children?
- Nutritional discharge criterion is currently parenthesised as “based on lack of oedema or MUAC >=12.5cm”. Would the authors please check – I think both resolution of oedema *and* weight thresholds are required.
- Randomisation and blinding: In terms of blinding, please clarify “laboratory tests are to be performed in a blinded manner” – who is being blinded, and how? Given that anthropometry is important to the outcome ascertainment, who will perform this and is there any blinding possible here? It would be helpful to know the method of randomisation e.g. is there a pre-specified block allocation?
- RUFT should be corrected to RUTF in the abbreviations section.
- There is a “X” indicating randomisation at day of admission in Table 1 – I think this may be an error.

Is the study design appropriate for the research question?

Yes

Is the rationale for, and objectives of, the study clearly described?

Yes

Are sufficient details of the methods provided to allow replication by others?

Yes

Are the datasets clearly presented in a useable and accessible format?

Not applicable

Reviewer Expertise:

I am an adult general physician and clinical researcher with experience of cohort studies and RCTs in sub-Saharan Africa.

I confirm that I have read this submission and believe that I have an appropriate level of expertise to confirm that it is of an acceptable scientific standard, however I have significant reservations, as outlined above.

# Response

**Published:** 2021-10-01

**Authors:** Kathryn Maitland

**Affiliations:** KEMRI-Wellcome Trust Research Programme, Kenya

Thank you Dr Rylance for the very detailed and helpful review.

 I have clarified our scope of health economic assessment and agree cost effectiveness does seem to be too definitive and therefore I am amended accordingly. 

 I have added details on the consent process, added a section on SAE reporting, clarified how neurocognitive outcomes will be assigned; added that the sickle cell genetic results will be send to the PI for communication to the families; added more details on contacts for data sharing.

**Minor issues**

 I have added the reference for definition of VSP (and included in the inclusion criteria) the details of the criteria. I have added a reference to the recommendations for 175-200cal/kg/day; amended the nutritional discharge criterion to MUAC> 12.5 AND lack of oedema. I have added in more details to who is blind to randomisation and that the clinic visits are conducted by the study team who are aware of the randomisation group. Included in the statistical section is more details on computer generated block randomisation.

# Peer review: Reviewer response for version 1

**DOI:** 10.21956/wellcomeopenres.18914.r45742

**Published:** 2021-09-10

**Authors:** Carina King

**Affiliations:** 1 Global Public Health, Karolinska Institute, Stockholm, Sweden

The COAST-Nutrition trial protocol lays out an interesting, and relevant study, to try and understand how we can improve survival in children with severe pneumonia. I had a few comments for clarifications in the methods presented:

- In the sample size, the trial registration indicates that 2200 children were originally planned. Could the change from that to 840 be expanded on? 
- Randomisation: how are the sealed opaque envelopes distributed from Kilifi to the other three study sites securely? And was the 1:1 stratified by site, or overall? 
- In the ISRCTN trial registration, it mentions that participants are children taking part in the COAST trial. Were all study arms of this trial eligible?
- And following on, was consent for the two different trials taken separately, with the opportunity to take part in one but not the other? The explanation of how the two trials are linked in the discussion section was super helpful, and it might help clarify this aspect if this appears earlier on in the paper. 
- Could more information on how the RUTF is supplied be given (i.e. are caregivers given all 56 days worth of RUTF on discharge, or are they required to return to the hospital to pick it up). Is adherence to feeding in the intervention arm and caregiver/child experiences/perspectives measured?
- Information on how standardisation of clinical and anthropometric assessments at recruitment and follow-up was done across the different sites would be helpful (e.g. training, quality checks, data cleaning).
- In the follow-up assessments, is the assessor blinded to the allocation status of the child?
- I didn't quite understand how the change in MUAC (continuous?) would be combined with mortality (binary?) into a single composite primary outcome variable. Apologies if I've misunderstood this. 

Minor edits:

- Abstract: I think "cost-economic analysis" might be a typo?
- Introduction: "Relevant to the question COAST-Nutrition trial seeks to address is children hospitalised with clinical diagnosis of very severe pneumonia (VSP), who had the second highest hazard ratio for post-discharge mortality (after malnutrition) of 4.1 (95%2.3-7.5)" a few words are missing in this sentence. 
- Introduction: remove "ration 1:1" in the aim statement at the end.
- Objective (iii): is not written as an objective. 
- Objective (iv): can you be more specific with the "overtime" component of this objective? I presume day 90 and 180?
- Inclusion criteria: it would be helpful to explicitly list the signs of severe pneumonia used, particularly to be clear on whether severely malnourished children classified by WAZ but not MUAC would be excluded.
- For the ages, in Figure 1 you have >6 months to <12 years, but in the text, you have between 6 months and 12 years, so it is not 100% clear if 6 months olds and 12 year olds are included. 
- Outcomes: typo in the statement "Re-admission to hospital by 28 and day 180 days"

Is the study design appropriate for the research question?

Yes

Is the rationale for, and objectives of, the study clearly described?

Yes

Are sufficient details of the methods provided to allow replication by others?

Partly

Are the datasets clearly presented in a useable and accessible format?

Not applicable

Reviewer Expertise:

Epidemiologist

I confirm that I have read this submission and believe that I have an appropriate level of expertise to confirm that it is of an acceptable scientific standard, however I have significant reservations, as outlined above.

# Response

**Published:** 2021-10-01

**Authors:** Kathryn Maitland

**Affiliations:** KEMRI-Wellcome Trust Research Programme, Kenya

Thank you Dr King for a very helpful and detailed review.

 With respect to the sample size changing I have included in the relevant section why the sample size was revised as the MUAC change of 0.5 was thought to be more clinical relevant.

 The 1:1 randomisation was stratified by site in block randomisation. The randomisation envelopes were prepared in Kilifi in batches and then sent by courier to the respective sites. Envelopes had a signature over the seal and could not be used if the seal had been tampered with or broken.

 Yes all children except those < 6 months of age and those with severe malnutrition were included in the factorial trial. Initially children were randomised to oxygen strategies then at 48 hours eligible children randomised to nutritional strategies. The oxygen trial was terminated (detailed in the protocol manuscript) and the protocol was amended so children are only randomised to nutritional strategies. 

 The consent and patient information sheets are included in the supplemental files. All children were eligible for both trials (see above) except those with were excluded for age (children 6 months and below are meant to exclusively breast fed) and children with malnutrition. 

 I have included in the section on nutritional feeds how we supply these in the trial. We are not collecting details on whether the supplement is being taken by the child and experiences/perspectives. 

 All the study centres use a detailed manual of operations, together with pre and peri trial training which includes a detailed section on how to assess the anthropometric measures. All data are double data entered and discrepancies resolved with trial managers.

 I have now specified in the protocol that at follow up children are assessed by the clinical team who are aware of the nutritional randomisation. 

 For the co-primary endpoint death (bivariate outcome) is treated as the worse scenario as a competing outcome with change of MUAC from admission to Day 90 (which is continuous measure) 

**Minor comments**

 Thank you for flagging typos and confusing sentences. These have all been addressed.

 Included in the inclusion criteria I have added as a foot note the definitions of severe pneumonia from the WHO Pocket Handbook for inpatient management. 

 I have also clarified children over (>) 6 months are eligible to children aged 12 years.
